# Overcoming beta-lactam resistance in *Pseudomonas aeruginosa* by targeting metallo-beta-lactamase VIM-1: a one-microsecond molecular dynamics simulation study

**DOI:** 10.3389/fcimb.2025.1521391

**Published:** 2025-02-04

**Authors:** Mohammed Salleh M. Ardawi, Samar A. Badreddine, Muhammad Yasir, Aiah M. Khateb, Safaa A. Turkistani, Ahmed Afandi, Samah O. Noor, Adhari Alselmi, Vivek Dhar Dwivedi, Esam I. Azhar

**Affiliations:** ^1^ Department of Pathological Sciences, Fakeeh College for Medical Sciences, Jeddah, Saudi Arabia; ^2^ Infection Control Department, Dr. Soliman Fakeeh Hospital, Jeddah, Saudi Arabia; ^3^ Special Infectious Agents Unit, King Fahd Medical Research Center, King Abdulaziz University, Jeddah, Saudi Arabia; ^4^ Department of Medical Laboratory Sciences, Faculty of Applied Medical Sciences, King Abdulaziz University, Jeddah, Saudi Arabia; ^5^ Department of Clinical Laboratory Sciences, College of Applied Medical Science, Taibah University, Medina, Saudi Arabia; ^6^ Medical Laboratory Sciences, Fakeeh College for Medical Sciences, Jeddah, Saudi Arabia; ^7^ Diabetic Foot Wound Center, King Fahad Armed Forces Hospital, Jeddah, Saudi Arabia; ^8^ Department of Biological Sciences, Faculty of Science, King Abdulaziz University, Jeddah, Saudi Arabia; ^9^ Clinical Sciences Department- MBBS Program, Fakeeh College for Medical Sciences, Jeddah, Saudi Arabia; ^10^ Dr. Sulaiman Fakeeh Medical Center, Jeddah, Saudi Arabia; ^11^ Center for Global Health Research, Saveetha Institute of Medical and Technical Sciences, Saveetha Medical College and Hospitals, Saveetha University, Chennai, India; ^12^ Bioinformatics Research Division, Quanta Calculus, Greater Noida, India

**Keywords:** *P. aeruginosa*, metallo-beta-lactamase, VIM-1, beta-lactam antibiotics, drug discovery

## Abstract

*Pseudomonas aeruginosa (P. aeruginosa)* is a Gram-negative opportunistic pathogen with a high resistance to beta-lactam antibiotics, mainly due to the production of metallo-beta-lactamase VIM-1 (MBL-VIM-1) enzyme. This study aimed to identify new inhibitors targeting MBL-VIM-1 to restore the efficacy of beta-lactam antibiotics. Extensive screening of natural compounds from the COCONUT database was performed to identify the structural analogs of the existing inhibitor of the MBL-VIM-1 protein. The virtual screening process selected four top-performing compounds (CNP0390322, CNP03905695, CNP0079056, and CNP0338283) that exhibited promising docking scores. These compounds were then subjected to re-docking and one-microsecond molecular dynamics (MD) simulations to assess their binding stability and interactions within the MBL-VIM-1 active site. Finally, post-MD simulation calculations were employed to estimate the interaction strengths and compare the efficacy of these compounds against the reference inhibitor. The findings highlighted that these four potent MBL-VIM-1 inhibitors show superior binding affinity and stability, suggesting their potential to combat antibiotic resistance in *P. aeruginosa*. The identified compounds offer a promising avenue for developing novel therapeutics to restore the efficacy of beta-lactam antibiotics against resistant bacterial strains. Therefore, further *in vitro* and *in vivo* studies are warranted to validate their potential.

## 1 Introduction

*Pseudomonas aeruginosa* (*P. aeruginosa*) is an encapsulated Gram-negative bacterium that plays a leading role in nosocomial infections. It mainly prevails among immuno-compromised persons and chronic disease patients (Markou and Apidianakis, 2014; Wood et al., 2023). This rod-shaped, aerobic bacterium is highly resilient in the adverse conditions of the host’s immune system. These characteristics facilitate versatile tropism, allowing them to colonize different human body sites, for instance, in the respiratory and urinary tracts (Kerr and Snelling, 2009; Wilson and Pandey, 2024). Due to its metabolic versatility, this bacterium also thrives in soil, water, and various surfaces within hospital environments, making it challenging to control and contain. Epidemiologically*, P. aeruginosa* has been proven to be associated with several outbreaks worldwide, mainly attributed to its resistance to several antimicrobial agents (Kowalski et al., 2001; Pachori et al., 2019; Schärer et al., 2023). These outbreaks have been especially catastrophic in the Intensive Care Units (ICUs) because the bacterium quickly causes ventilator-associated pneumonia, bloodstream infections, and surgical site infections among immune-compromised patients (Esposito and Leone, 2007). Moreover, the emerging MDR strains of this bacterium have worsened the situation with high morbidity, mortality rates, and treatment costs (Kunz Coyne et al., 2022).

Another key point in the pathogenicity of *P. aeruginosa* is its extensive array of virulence factors and mechanism for evading the host immune system and resisting antibiotic actions (Drenkard, 2003). Some of the significant factors are biofilm formation, efflux pumps that expel antibiotics, and beta-lactamases that degrade beta-lactam antibiotics into inactive small molecules (Dreier and Ruggerone, 2015) Beta-lactamase significantly contributes to antibiotic resistance by hydrolyzing the beta-lactam ring in many antibiotics, such as penicillins, cephalosporins, and carbapenems (King et al., 2014; Akhtar et al., 2022; Lin and Kück, 2022). Several types of beta-lactamases are produced by *P. aeruginosa*; however, Metallo-beta-lactamase (MBLs) are of particular concern. These enzymes that depend on divalent cations, such as zinc ions, for their function can cleave a large of types of beta-lactam rings from different groups of antibiotics that are usually used in the treatment of MDR bacterial infections (Tamilselvi and Mugesh, 2008; Karsisiotis et al., 2014).

The emergence of MBLs, particularly the Verona integron-encoded metallo-beta-lactamase (VIM) family, has been a significant driver of carbapenem resistance in *P. aeruginosa*. VIM-1, the first member of this family to be identified, was initially reported in Italy in the late 1990s and has since been detected in various parts of the world (Makena et al., 2016). The spread of VIM-1 is often associated with mobile genetic elements such as plasmids and integrons, which facilitate its dissemination across different bacterial species and strains.

The VIM-1 enzyme has been reported to be able to hydrolyze carbapenems. It is resistant to inhibition by the most commonly used beta-lactamase inhibitors, putting a serious challenge to managing infections produced by VIM-1-producing *P. aeruginosa* (Salimraj et al., 2019). As a result, the search for effective inhibitors against VIM-1 has emerged as one of the significant priorities of antimicrobial science. In particular, the action against MBL, like VIM-1, might present a good strategy to increase the efficacy of beta-lactam antibiotics against *P. aeruginosa*-resistant strains (Tehrani and Martin, 2017). Preliminary work towards discovering inhibitors for MBL VIM-1 has been undertaken, and several molecules have been identified; however, most show drawbacks. For instance, thiol-based compounds include thiomandelic acid, pharmacokinetic profile, and toxicity effects (Mollard et al., 2001). Other inhibitors, such as some hydroxamates and dithiocarbamates, also need help regarding specificity and stability. However, there are no clinically approved VIM-1 inhibitors, so it becomes necessary to find other ways around the constraints of the above-named candidates (Ju et al., 2018).

The COCONUT database reflects an enormous field of natural products containing possible pharmacological effects (Sorokina et al., 2021; Nainala et al., 2024). COCONUT has more than 6 lakh unique natural compounds and a broad chemical space coverage, vital for drug discovery and identifying new inhibitors to different targets, including MBL VIM-1. Since many of the compounds in the COCONUT database are of natural origin, they generally exhibit novel structural characteristics and bioactivities that have not been observed in synthetic libraries, thereby making them ideal sources for identifying lead compounds (Chávez-Hernández et al., 2020).

In this study, our objective was to find the potential inhibitors of MBL VIM-1 through the comprehensive analysis of the natural compounds obtained from the COCONUT database. Herein, a multi-step drug discovery protocol was employed. This includes virtual screening for filtering out the compounds using the Lipinski rule of five; molecular re-docking studies for verification and refinement of the selected compounds and target protein; molecular dynamic (MD) simulation to evaluate the stability and behavior of the generated complexes and molecular mechanics with Generalized Born and Surface Area (MM/GBSA) analysis to calculate the free energy of binding. Through this meticulous workflow, we identified natural compounds with substantial inhibitory potential against MBL VIM-1 protein, offering promising leads for developing new therapeutic strategies to fight MDR strains of *P. aeruginosa.*

## 2 Methodology

**Figure 1:**
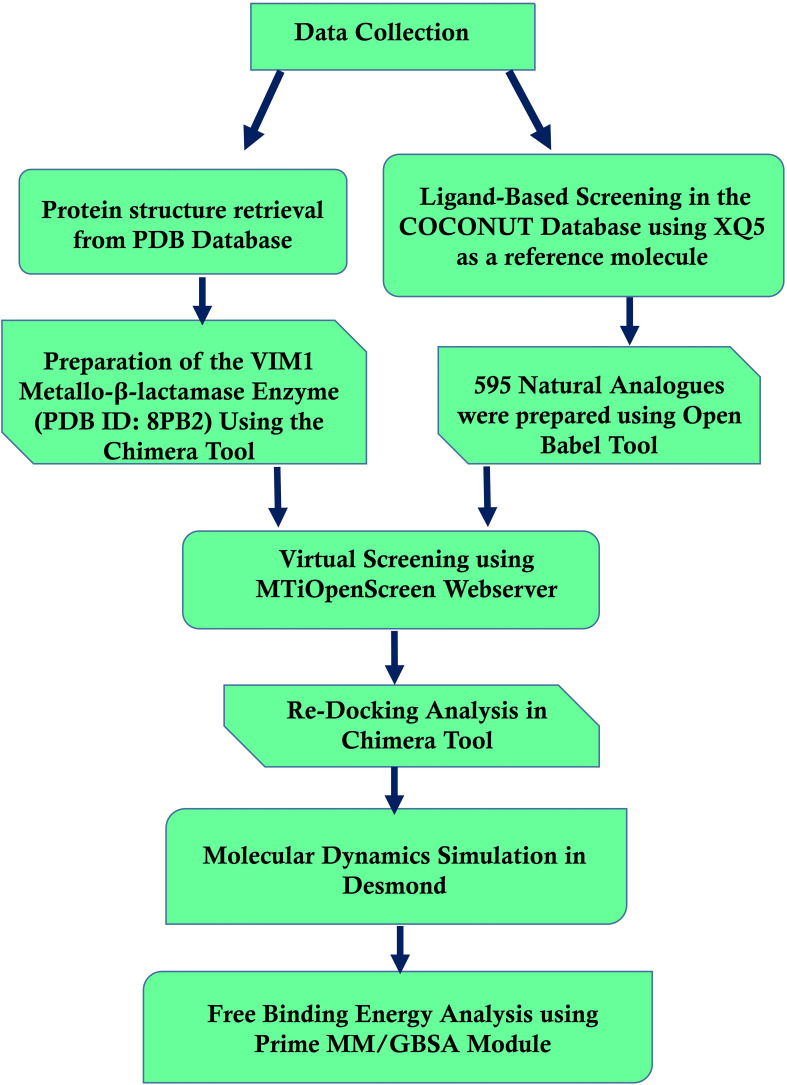
Workflow of computational methods for identifying *Pseudomonas aeruginosa* Metallo-Beta-Lactamase VIM-1 inhibitors.

**Figure 1** shows the workflow chart summarizing the computational approach used, including preparing protein and ligand, virtual screening, re-docking, molecular dynamics simulations, and free binding energy calculations. This flowchart acts like a map for the detailed methodology that follows, elaborating each step in detail.

### 2.1 Protein preparation and ligand data collection

The crystal structure of the target protein, MBL VIM1, was obtained from the Protein Data Bank (PDB ID: 8PB2) (Berman et al., 2000). The protein was prepared using the Chimera Dock Prep tool (Pettersen et al., 2004). This included the steps of hydrogen adding and charging according to the specified partial charges, as well as the elimination of water molecules and bound ligands to bring the protein to a state suitable for docking.

The COCONUT database containing 406,076 compounds was utilized for identifying the structural analog of the existing inhibitor XQ5 [(7-[(1~{S})-1-[4-(carbamimidamidomethyl)-1,2,3-triazol-1-yl]ethyl]-3-[3-fluoranyl-4-(methylsulfonylmethyl)phenyl]-1~{H}-indole-2-carboxylic acid)] present in the MBL VIM1 protein structure at a 70% Tanimoto similarity threshold (Mollard et al., 2001). The ligand structures were obtained from this database in SDF format and optimized to PDBQT format using Open Babel (O’Boyle et al., 2011). The natural compounds collected were prepared to maintain the protonation state using the Chimera tool (Pettersen et al., 2004; Trott and Olson, 2010). To further filter the input and select only drug-like molecules, the Lipinski Rule of Five was implemented (Lipinski, 2004), which acts on the compounds according to their physicochemical characteristics and then ensures that the compounds possess the desired pharmacokinetic traits.

### 2.2 Virtual screening and re-docking analysis

Virtual screening was performed using the MtiOpenScreen webserver (Labbé et al., 2015). For the screening process, a docking grid was precisely set at coordinates (X = -23.39, Y = -12.39, Z=10.76) and extended 30 Å along each axis (X, Y, Z). Further, the highest binding energy compounds obtained after the intensive screening along with the existing inhibitor XQ5 were again subjected to molecular re-docking using the AutoDock Vina plugin within UCSF Chimera (Trott and Olson, 2010). Re-docking involved generating multiple binding poses for each compound, allowing us to explore their interactions within the VIM1 active site more thoroughly. The AutoDock Vina algorithm was employed to refine the docking results, confirming the selected compounds’ binding affinities and interaction profiles. This re-docking process validated the initial screening results and provided a robust foundation for subsequent molecular dynamics simulations and free energy calculations. The XQ5 inhibitor was considered as a reference molecule for the comparative analysis with the selected natural compounds.

### 2.3 Molecular dynamic simulation and trajectory analysis

The MD simulations were conducted using the Desmond-maestro interpolarity tool free academic version, which is well-suited for studying the dynamic behavior of biomolecular systems (Bowers et al., 2006). To generate each of the protein-ligand complexes, we placed the system in an orthorhombic water box in such a way that the minimum distance between the solute and box periphery was 10 Å. The background charge of the system was also maintained at a low level by adding counter ions, thus mimicking biological conditions. The biomolecular force field OPLS-2005 was used for the parameterization of the protein and the ligands since this force field is reasonable and accurate for biomolecular interactions (Harder et al., 2016). Subsequently, energy minimization was conducted to eliminate any unfavorable close contacts in the generated system (Jaidhan et al., 2014). This process was crucial in achieving a steady state before the production phase of the simulation work. The systems were minimized and then gradually coupled with the temperature reservoir at 300 K for 100 picoseconds in the NVT ensemble. Equilibration was then carried out under the condition of several particles, pressure, and temperature (NPT ensemble) for 1 ns to give the system time to reach density and other thermodynamic properties before the actual production runs. The MD production simulations for all the complexes were performed for 1000 ns under the NPT ensemble only. Choose a time step of 2 fs and control the long-range electrostatic effect using the Particle Mesh Ewald (PME) method. Additional trajectories were obtained every 10 ps from the simulation process to facilitate a more refined resolution. Trajectory analysis concentrated on the evaluation of the stability and changes in the protein-ligand structure. The structural strength of the protein backbone and flexibility of the involved residues were assessed using Root Mean Square Deviation (RMSD) and Root Mean Square Fluctuation (RMSF) of the final simulation frames. The number of hydrogen bonds between the protein and the ligand was recorded during simulations to capture the stability of these bonds.

### 2.4 Free binding energy using MM/GBSA calculation

The free binding energy calculations were performed using the Prime MM/GBSA module (Schrödinger Release 2024-2: Prime, Schrödinger, LLC, New York, NY, 2024) to compare the binding energies of the chosen MBL VIM1 inhibitors. Assigning the free binding energies, the MMGBSA method helped to obtain a considerable number of data regarding the interaction constancy and intensity between the ligands and the VIM1 protein (Genheden and Ryde, 2015a). Using the Generalized Born model, the MMGBSA method calculated the binding free energy through molecular energies and solvation. This method allowed the evaluation of enthalpy and entropy associated with the binding event when calculating the binding free energy. The binding energies were thus calculated and compared between the four complexes as well as the reference molecule, in terms of their ability to inhibit VIM1 quantitatively. This detailed analysis provided key insights into the most promising inhibitors for further experimental validation.

## 3 Results

### 3.1 Virtual screening analysis

A total of 595 natural compounds, structurally analogous to the native inhibitor XQ5, were identified and selected for structure-based virtual screening. Of these, 332 compounds were filtered out, exhibiting energy ranges between -10.816 kcal/mol and -3.129 kcal/mol, as detailed in **Supplementary Table S1**. Four natural compounds—CNP0390322, CNP0390569, CNP0079056, and CNP0338283—were selected for further analysis based on their docking energies, which were -10.81 kcal/mol, -10.79 kcal/mol, -10.76 kcal/mol, and -10.73 kcal/mol, respectively. While according to ADME’s available data, these selected compounds exhibit superior drug-like properties compared to the control, they have lower molecular weights, higher lipophilicity (higher XLogP3 values), and smaller topological polar surface areas, which indicate better pharmacokinetics and bioavailability. Additionally, these natural compounds are simpler and more defined chemical structures than the native inhibitor XQ5 suggesting more selective biological interactions, making them stronger candidates for further study.

### 3.2 Redocking and intermolecular calculation

**Figure 2:**
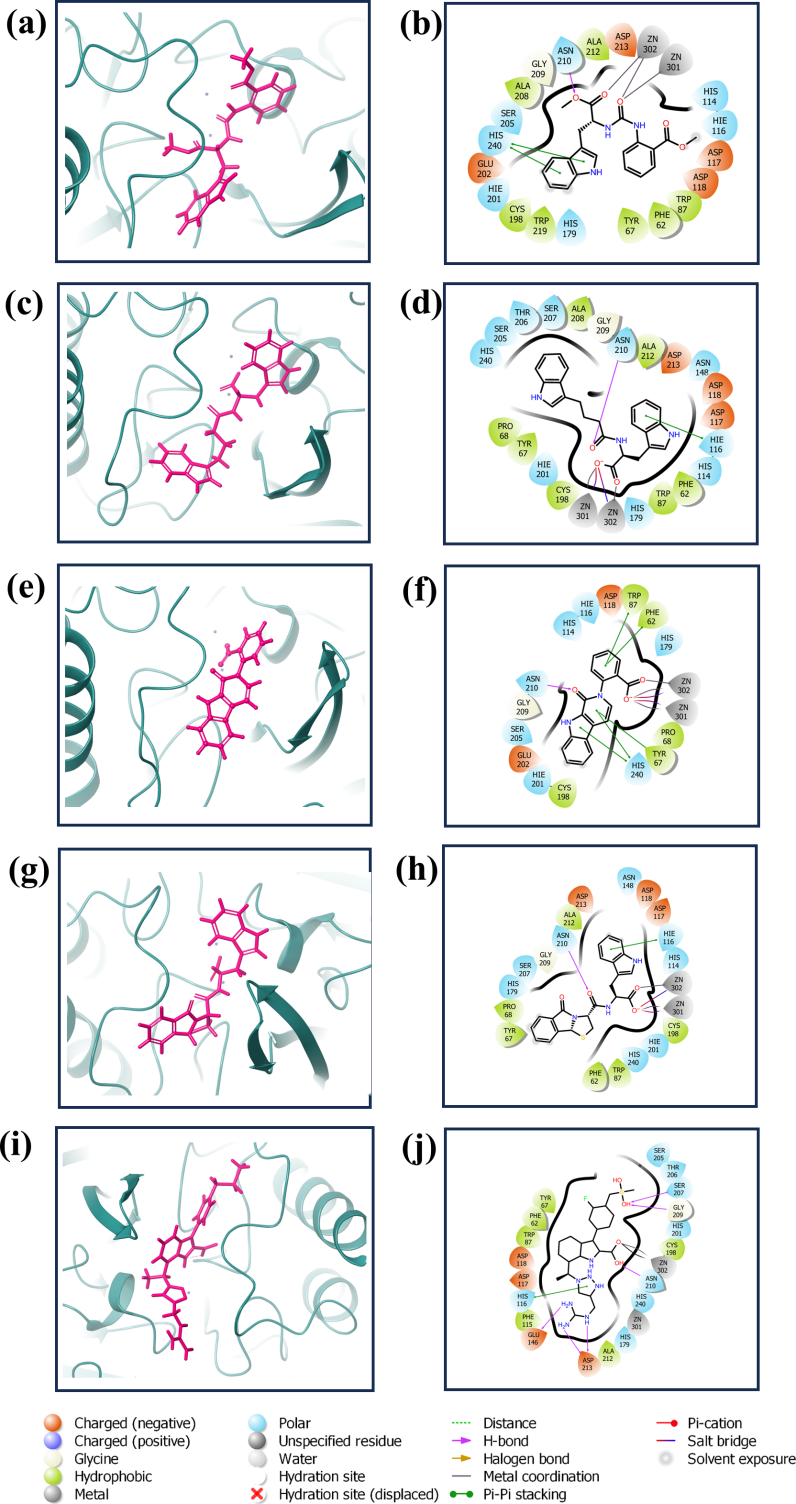
3D and 2D interaction diagram of the VIM1 docked with selected compounds: **(A, B)** CNP0390322, **(C, D)** CNP0390569, **(E, F)** CNP0079056 **(G, H)** CNP0338283 and **(I, J)** XQ5 (reference).

The redocking procedure was conducted for the four selected natural compounds that exhibited the highest negative docking scores from our virtual screening, aiming to form stable complexes with the target protein. The redocking results underscored the robust binding potential of these compounds to the target protein, affirming their suitability for more detailed examination. The calculated binding energy of selected compounds (1, 2, 3, and 4) was -8.7 kcal/mol, -8.7 kcal/mol, -9.4 kcal/mol, and -9.6 kcal/mol, respectively, and -10.7 kcal/mol for the control (XQ5). This method not only validated the initial virtual screening results but also confirmed that the selected compounds are strong candidates for subsequent studies, as shown in **Figure 2**. The 3D and 2D structures were generated using Maestro, as shown in **Figure 2**.

The complex VM1-CNP0390322 exhibited a strong interaction profile characterized by several key interactions. The complex formed a hydrogen bond with Asn210, a residue commonly engaged in stabilizing ligand binding through hydrogen bonding networks. In terms of hydrophobic interactions, the VM1-CNP0390322 complex was surrounded by several hydrophobic residues, including Phe62, Tyr67, Trp87, Cys198, Ala208, Ala212, and Trp219, which contribute to the stabilization of the ligand within the hydrophobic pocket of the protein. Additionally, a significant π-π stacking interaction is observed with His240, indicating a potential role in further stabilizing the ligand through aromatic interactions. The compound CNP0390569 in the target protein also interacts with Asn210 via hydrogen bonding, indicating the importance of this residue in ligand stabilization. Hydrophobic interactions are observed with Phe62, Tyr67, Pro68, Trp87, Cys198, Ala208, and Ala212. These residues form a hydrophobic pocket’s favorable condition for ligand binding—the interaction between ligand and His116 in this complex forms π-cation for stabilizing the binding of the protein. Likewise, CNP0079056, when complexed with VM1, builds another hydrogen contact with Asn210, and this residue contributed a similar role among various complexes. The hydrophobic interactions have been formed by Phe62, Tyr67, Pro68, Trp87, and Cys198 residues; they are conserved in all three complexes. Among the three, this complex exhibits multiple π-π stacking interactions with Phe62, Tyr67, Trp87, and His240, suggesting a stronger aromatic interaction pattern that may improve the stability and the interactions between the ligand and amino acids. The VM1-CNP0338283 complex showed a hydrogen bond with Asn210, strengthening the ligand anchoring hypothesis. These involve Phe62, Tyr67, Pro68, Trp87, Cys198, and Ala 212, present in a homologous conserved hydrophobic pocket seen in the prior complexes. It is, therefore, possible to conclude that the π-cation interaction with His116 describes a similar binding mode to that of the VM1-CNP0390569 complex. In the VM1-XQ5 control complex, multiple hydrogen bonding was formed by Glu146, Ser207, Gly209, Asn210, and Asp213. The Phe62, Tyr67, Trp87, Phe115, Cys198, and Ala212 residues aided the ligand in fitting into the pocket through the hydrophobic bond. The presence of the π-cation interaction with His116, characteristic of all other complexes, suggests that these ligands have similar binding profiles. The behaviors of these complexes revealed that Asn210 is critical for hydrogen bonding while hydrophobic pocket conserved by Phe62, Tyr67, Trp87, and Cys198, and π-π stacking or π-cation interaction with His116 and His240 in stabilizing the ligand-protein complex (**Supplementary Table S2**).

### 3.3 Structure-activity relationship analysis of compounds interacting with VIM-1

The analysis of the interactions between the compounds and VIM-1 reveals critical structure-activity relationships. Hydrogen bonding with Asn210 is a consistent feature across all compounds, indicating its pivotal role in ligand stabilization within the binding pocket. Additionally, hydrophobic interactions with residues such as Phe62, Tyr67, Trp87, Cys198, Ala208, and Ala212 form a stable hydrophobic pocket, which supports the anchoring of the ligands. The presence of π-π stacking and π-π cation interactions with His240 and His116 highlights the importance of aromatic moieties in the compound structures, enabling favorable interactions with key aromatic residues in VIM-1. These findings underscore the significance of hydrogen bond donors/acceptors, hydrophobic substituents, and aromatic centers in driving the ligand’s affinity and specificity for VIM-1.

To optimize binding, future modifications could enhance hydrogen bonding with Asn210 and strengthen interactions within the hydrophobic pocket by introducing suitable non-polar substituents. Retention or expansion of aromatic rings would further leverage π-π stacking interactions, particularly with His240 and His116. These insights can be experimentally validated through mutational studies targeting Asn210, His116, and His240 to confirm their functional roles, alongside testing derivatives with modified chemical groups.

### 3.4 Dynamical analysis

In our study, the stability and flexibility of the four complexes were investigated by 1000 ns MD simulations. These simulations were very important in observing the dynamic behavior of the complexes. The key parameters like RMSD and RMSF were carefully monitored and recorded for protein and ligand parts. RMSD measurements were helpful in analyzing the general stability because they presented their complex structures as a function of time in terms of deviations from their starting conformations. Conversely, RMSF analysis showed the movement of individual protein residues that can be affected in terms of the structural changes that might affect ligand binding. Further, the formation and duration of hydrogen bonds in each complex and throughout the entire simulation period were also observed to determine the interactions involved in the complexes’ stability.

#### 3.4.1 RMSD analysis

**Figure 3:**
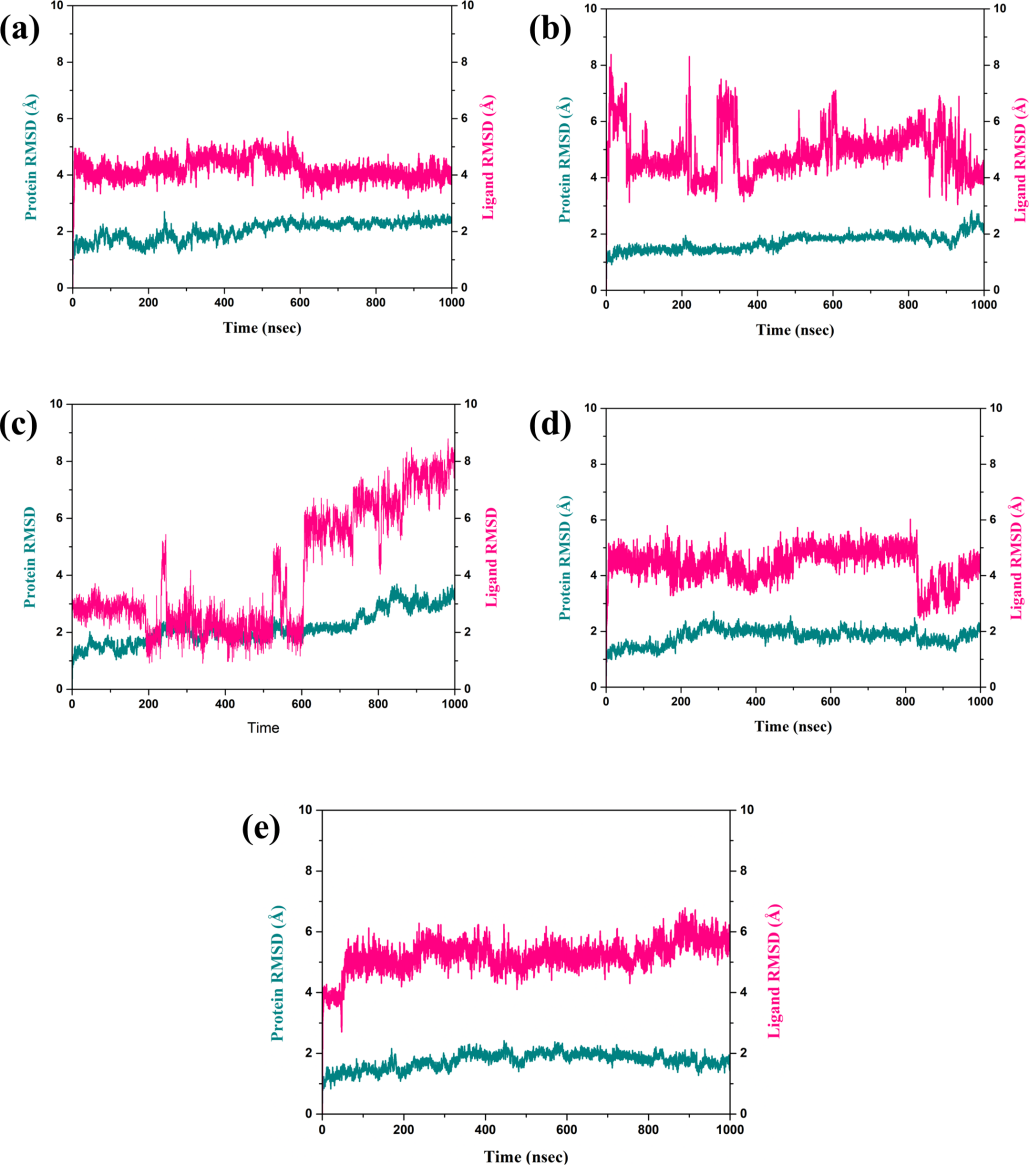
RMSD plot of the simulated VIM1-natural compounds **(A)** CNP0390322, **(B)** CNP0390569, **(C)** CNP0079056, **(D)** CNP0338283 and **(E)** XQ5 (reference).

The RMSD is a very important parameter for measuring the stability of a complex during MD simulations. They bring information about conformational changes and the general stability of the protein and the ligand over time, as depicted in **Figure 3** (Pavan et al., 2022). Thus, in this analysis, the fractional RMSD of the ligand has been seen along with the protein dared over a simulation time of 1000 ns of four compounds also one control molecule. The backbone RMSD for the protein in the compound CNP0390322 in complexed with protein was stable with an average range of 1.5 Å to 2 Å for the last 100 ns of simulations out of 1000 ns of simulation period. This means that the protein is conservative in its conformation suggesting a good structural stability during its interaction with CNP0390322. The RMSD of the ligands oscillates in a range between 4 Å and 4.5 Å and occasionally reaches values above 5 Å but tends to stabilize in the further course of the simulation. Fluctuations in the protein RMSD and the ligand RMSD minimally suggest that compound CNP0390322 has a stable binding mode within the active site, and the conformation change of the protein during the simulation is minimal. The protein RMSD in the compound CNP0390569 in complex with protein was similar to that of compound CNP0390322, which varied from 1.5 Å to 2.0 Å. However, these values reach a fixed, stable point after 200 ns, implying that the protein might alter its conformation slightly and then attain a more stabilized state. The ligand RMSD was more variant and oscillated between 2.0 Å and 8.0 Å, and the variations were even more prominent in the first 200ns but gradually settled at about 5. Å. Higher RMSD values arising from compound CNP0390569 suggest more substantial conformational flexibility within the binding pocket and, hence, a less stable binding profile as compared to compound CNP0390322. The protein RMSD for CNP0079056 in complex with protein deviated from 1.5 Å to 2.0 Å. The target protein remains reasonably stable throughout the simulation, indicating that the protein conformation does not change appreciably upon binding with the compound CNP0079056. The ligand RMSD fluctuates from the value of around 1.5 Å to 3.0 Å and increases somewhat with time but remains viable beyond 200 ns. Such a pattern implies that compound CNP0079056, maintains its localization within the binding pocket with insignificant changes, exhibiting high binding affinity and stability. For the compound CNP0338283 complex, the protein RMSD varies slightly from 1.5 Å to 2. Å then remains steady. The secondary structure of the protein seems to be reasonably good, indicating a proper orientation with the ligand. The range of the ligand RMSD in this complex varies from 2.0 Å to a maximum of 6.0 Å with occasional spikes. Nevertheless, these RMSD values reflect the fact that the ligand is also somewhat flexible within the binding pocket while the interaction is rather constant over time. The variation of the control molecule demonstrates the protein’s RMSD in a stable condition with values of 1.5 Å- 2.0 Å, as in the case of CNP0390322 and CNP0079056. This stability demonstrates that much of the protein’s structure remains rigid or unchanging. Comparing the RMSD of the control molecule for the ligand shows higher fluctuations, which vary between 5.0 Å to 6.0 Å and have a general tendency to increase as the simulation progresses. This implies that the control ligand rotated more freely and was less stable in the binding site of the protein.

#### 3.4.2 Protein RMSF analysis

**Figure 4:**
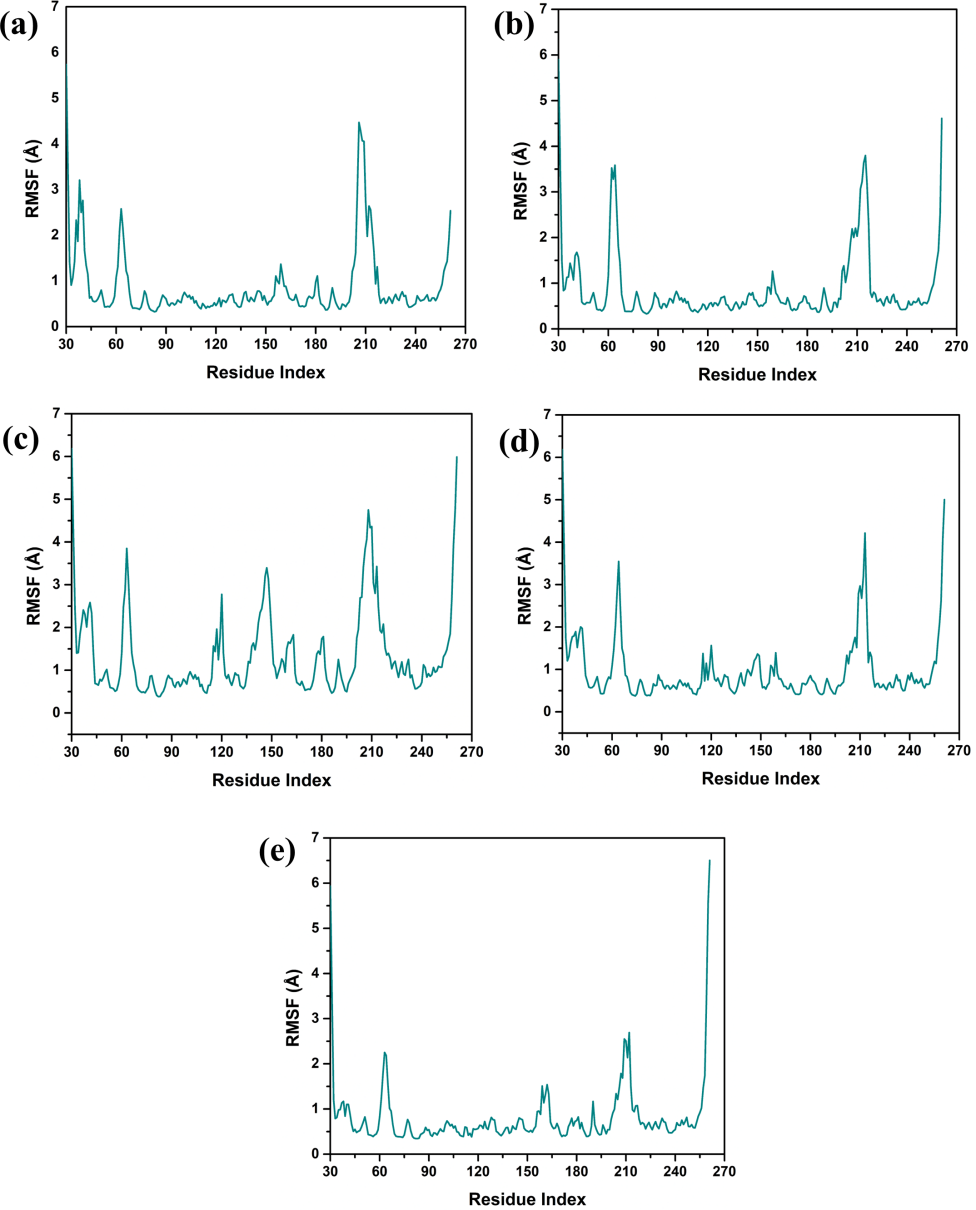
Protein RMSF plot of the simulated VIM1-natural compounds **(A)** CNP0390322, **(B)** CNP0390569, **(C)** CNP0079056, **(D)** CNP0338283 and **(E)** XQ5 (reference).

Structural flexibility and stability derived from RMSF proved valuable in determining the freedom and steadiness of specific residues within the complexes, as indicated in **Figure 4** (Sharma et al., 2021). The RMSF analysis was performed to compare the dynamic flexibility of the complexes in different compounds. RMSF is an atomic fluctuation from its average position when calculated for a set of atoms and its capability to analyze the dynamic behavior of certain protein parts compared to others during molecular dynamics simulations. In the case of the VM1 protein complex with compound CNP0390322, the RMSF showed a value of 4.5 Å within the range of amino acid positions 170 – 190. This implies that this part of the protein shows moderate flexibility in complexation with compound CNP0390322, and this aspect can have an influence on the dynamics of the tightening interaction, including the binding strength of the formed complex. For the complex VM1-

CNP0390569, the protein showed an RMSF value of less than 4 Å within the same range of amino acids. This lower RMSF value suggests that the protein becomes more rigid when in the presence of CNP0390569, which may stabilize the binding conformation in this area. Like CNP0390322, CNP0079056 had an RMSF of 4.5 Å for the residues 170–190. This indicates that compounds CNP0390322 and CNP0079056 may cause similar flexibility in this area of the protein, most likely to affect binding and overall stability within a similar framework. On the other hand, the compound CNP0338283 has an RMSF value of less than 4.5 Å in the similar amino acid range, slightly less than compounds 1 and 3 but more flexible compared to CNP0390569. Lastly, in the control molecule XQ5, the RMSF value was below 4.5 Å as well, but this time in the region of amino acids 170 – 190. Comparability of this stabilization with the compounds seen in the control group to some of the other complexes could be seen due to the similar effect on the protein dynamics.

#### 3.4.3 Ligand RMSF analysis

**Figure 5:**
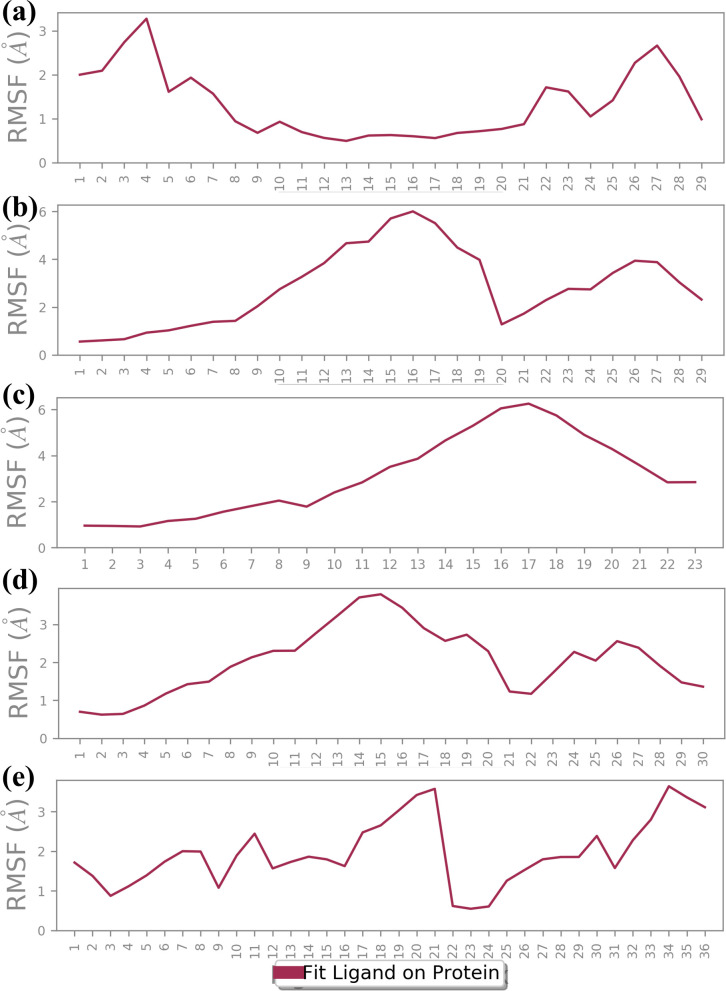
Ligand RMSF plot of the simulated VIM1-natural compounds **(A)** CNP0390322, **(B)** CNP0390569, **(C)** CNP0079056, **(D)** CNP0338283 and **(E)** XQ5 (reference).

RMSF analysis provides insights into the flexibility of the ligand atoms during the MD simulation as shown in **Figure 5**. Higher RMSF values indicate more flexible regions of the ligand, while lower values suggest more stable regions. The RMSF plot for CNP0390322 shows significant fluctuation, particularly around atom 5 and atom 27, with peaks reaching approximately 3.0 Å. The RMSF values of these peaks depict these regions of the ligand as flexible, while the other parts of the molecule have near-stable RMSF values of between 1.0-2.0 Å. The residue-specific profiles of CNP0390569 show a steady increase in flexibility up to the 17th atom with the RMSF value of about 6.0 Å. This means that a particular region within the ligand is more flexible during the simulations, suggesting that it may be flexible or have a substructure that is flexible in solution while the remainder of the ligand has a more rigid structure. The changes in the RMSF profile found for CNP0079056 are highest around atoms 9 up to 22, and the maximum is at about 6 Å. This alone points to the fact that the middle part of the ligand is relatively more mobile compared to the other regions where lower RMSF values hint at greater stiffness. Analyzing the CNP0338283, it is possible to observe that the RMSF values constantly increase from the beginning of the molecule and reach the maximum value between atoms 14 and 20 with approximately 3.5 Å. This suggests that the middle and towards the end of the ligand have a certain flexibility, and the remaining parts of the ligand remain almost rigid. On the other hand, control has the largest RMSF fluctuations with regard to the values across the molecule. Notable peaks occur around atoms 17 and 22, reaching up to 3.5 Å. This suggests that the control ligand is more flexible throughout the simulation, indicating weaker binding stability in comparison to the tested compounds. The RMSF analysis highlights the different flexibility profiles of the ligands across the compounds. Compounds 1 and 4 show specific regions of increased flexibility, while CNP0390569 and 3 demonstrate central region flexibility.

#### 3.4.4 Protein-ligand profiling

**Figure 6:**
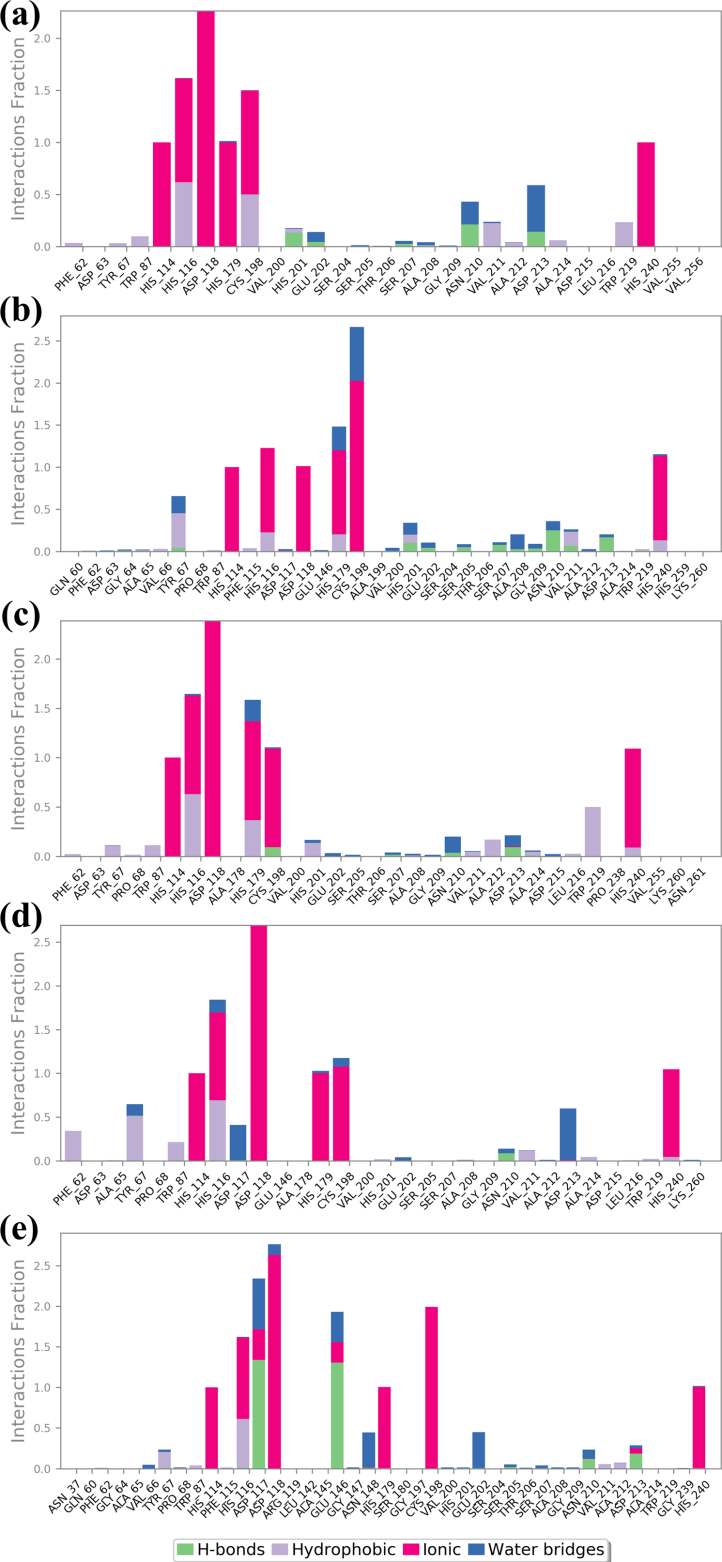
Protein-ligand profile analysis of the simulated VIM1-natural compounds **(A)** CNP0390322, **(B)** CNP0390569, **(C)** CNP0079056, **(D)** CNP0338283 and **(E)** XQ5 (reference).

Information about the interaction of protein and ligand is crucial in determining the stability and significance of ligand to the target protein. The understanding of various types of interactions, namely hydrogen bonds, hydrophobic interactions, cations and anions interactions, and water-mediated bridges, offers insight into the direction and strength of binding (Chen et al., 2016). The specific patterns of the observed interactions for each of the four compounds and the control are described in **Figure 6**. Ligand binding requires hydrogen bonds, especially for the specificity and stability of the interaction. Similarly in complex CNP0390322, VAL200 and ASN210 are greatly involved with hydrogen bonding with the ligand. These bonds presumably play a role in the enhancement of stability of the ligand within the matrix and strong interaction. In the present context, hydrophobic interactions assist in stabilizing the ligand so that it is shielded from water molecules in the binding site. PHE62 and TRP219 contribute significantly to the ligand molecule; both are hydrophobic in nature; it is indicated that these residues have an important role in tethering the ligand molecule firmly in the protein matrix. It also assists in maintaining the structural stability of the complex mainly because of the high-rise nature of this structure. Ionic interactions are mostly observed with basic and acidic residues, and the strength of the interaction is reflected in the binding energy. This is found to be present at positions such as HIS114, ASP118, and HIS240, a positively charged residue, hence participates in the ionic interactions or probably increases the binding strength of the providers to the ligands by direct electrostatic attraction. Few water bridges were observed, which also suggests that the most important interactions are direct contacts between the ligand and the protein. Compound CNP0390569 interacts with HIS201 and ASN210 through strong hydrogen bonds. These interactions may well be crucial for the positioning of ligands in the binding site and to the stability and possibly the efficacy of the compound. Whereas TYR69 and HIS116 give a hydrophobic pocket that stabilizes the ligand through nonpolar forces. These residues presumably help in positioning the ligand correctly and also in increasing the binding affinity by avoiding exposure of the ligand to water. The presence of ionic interactions between the TRP87, ASP118, HIS179, and CYS198 and the ligand point to the existence of electrostatic forces in further enhancing the binding affinity of the ligand into the binding pocket. The relative frequency of water bridges indicates that the ligand likely interacts directly with the binding site and indirectly through water molecules, which may give the binding site more versatility in terms of strength and flexibility. Like Compound CNP0390569, the present compound has also been observed to form intense H-bond interactions with both, CYS198 and ASP213. These interactions are significant for the stability and orientation of the ligand, indicating that the ligand has a rather high specificity for binding these residues. The ligand in Compound CNP0079056 has pronounced hydrophobic contacts with HIS116, HIS179, and TRP219 residues. These interactions help to maintain the stability of the complex because the correct orientation of the ligand in a binding pocket always prevents adverse forces like water from affecting the structure. These ionic interactions are HIS114, ASP118, HIS116, and CYS198, implying the compound’s consistent interaction type in stabilizing the ligand through ionic binding interaction, which supplements the binding interaction. This is particularly evident where water-mediated interactions indicate that direct contacts are stronger, but the ligand also uses water molecules to bridge interactions with the protein; a factor that could also contribute to the flexibility or adaptability of the binding. As in the case of the other compounds, CNP0338283 has strong interactions with ASN210 mainly by means of hydrogen bonds. Such a similar pattern observed in a number of compounds supports the view on the importance of these residues for the stabilization of ligands that have to remain bound to the protein with high selectivity. The hydrophobic interactions of PHE62, TYR67, and HIS116 are strongly indicative that all these residues are crucial to the stability of the ligand. These interactions are useful in sustaining the ligand at the correct orientation and position and may even improve the binding selectivity by reducing entropy changes during binding. The presence of interacting ionic residues such as HIS114, HIS116, ASP118, and HIS179 with the ligand supports the statement that electrostatic forces are vital in stabilizing the ligand-protein complex. The change in the number of water bridges present in CNP0338283 indicates that water molecules seem to play a more active role in the ligand and protein interactions; the water bridge may provide the ability to change conformation to accommodate for slight changes in the structures of the protein and ligand during the course of the simulation. The control ligand makes comparatively fewer hydrogen bonds than the test compounds that are mainly with ASP 117 and GLU 146 residues. This may indicate that the frequency of hydrogen bonding is less in this case and, therefore, might be less strong or less stable binding interactions than the other ligands. The control ligand also interacted with HIS 116, VAL 66, and HIS 116 in hydrophobic interactions, as observed with the test compounds. Nevertheless, these interactions are not as often, and that means that it could be much more challenging to stabilize the control ligand inside the binding pocket. While ionic interactions are observed with HIS114, ASP118, HIS179, and CYS198, the interaction fraction is less compared with test compounds. This could mean that the electrostatic interaction favored by the control ligand is not as strong, which yields a less stable complex. The control ligand again exhibited the most frequency of the water bridges. That such a significant amount of interactions are mediated by water might imply that the control ligand is not very firmly fixed in the binding pocket, and its binding can only be stabilized by weak transient water bridges.

**Figure 7:**
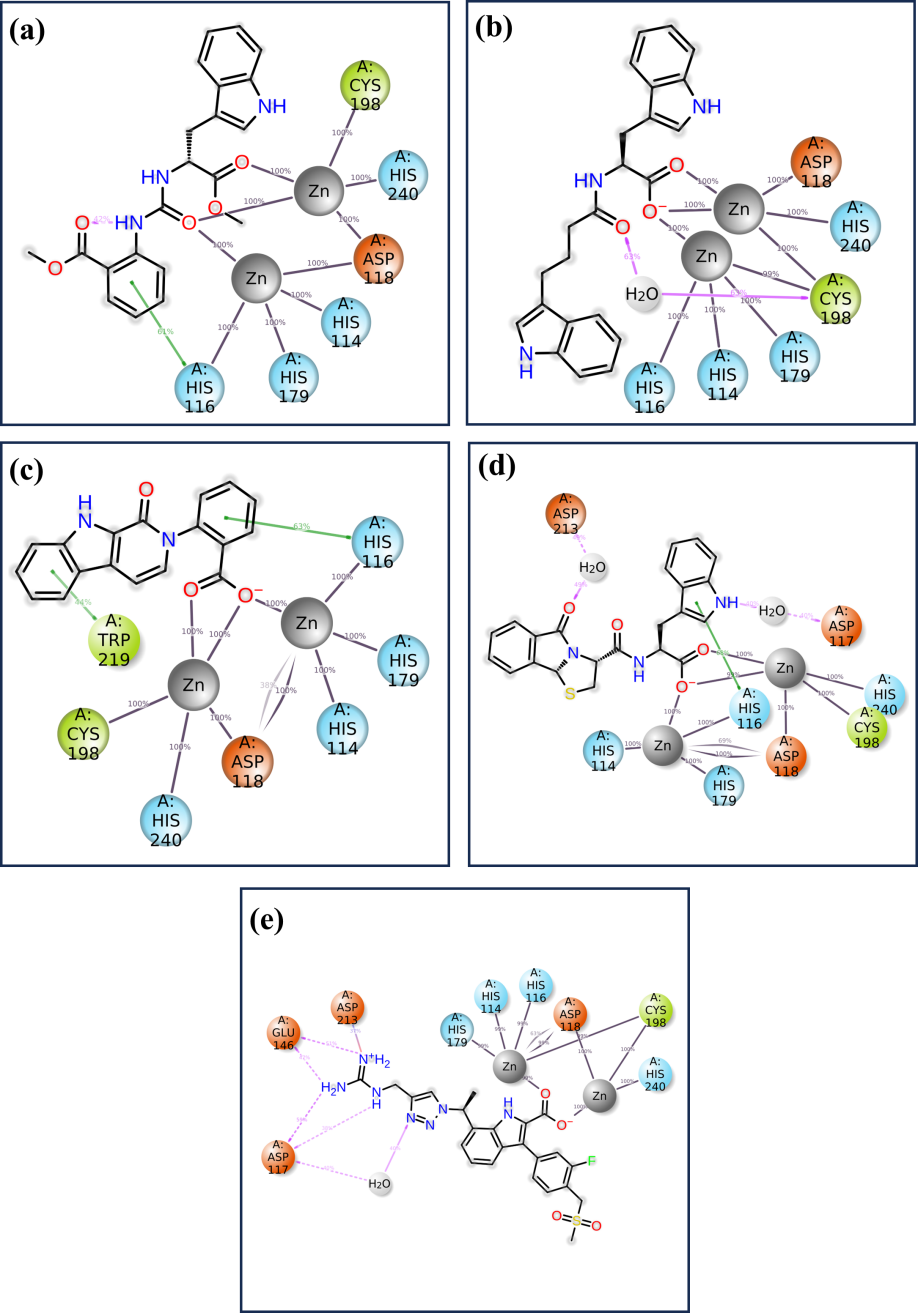
The 2D interaction of diagram of the simulated VIM1-natural compounds **(A)** CNP0390322, **(B)** CNP0390569, **(C)** CNP0079056, **(D)** CNP0338283 and **(E)** XQ5 (reference).

The detailed analysis of protein-ligand interactions for the four test compounds (1, 2, 3, and 4) and control reveals a complex and nuanced binding landscape within the active site of the target protein, as shown in **Figure 7**. The interactions were assessed based on their frequency of occurrence during molecular dynamics simulations, providing insights into the stability and nature of these interactions. Such analysis is crucial for understanding the binding efficacy and potential of these compounds as inhibitors or modulators of the protein’s function. Across all four test compounds, a consistent pattern of interaction was observed with key residues: HIS 116, HIS 114, HIS 179, CYS 198, HIS 240, and ASP 118. Each of these residues showed a 100% interaction frequency, indicating that they played a very important role in stability. The active zinc ion (Zn2+) also gave a 100% interaction frequency within all the compounds and plays a critical role in the coordination of the ligand via metal chelation. These interactions may be considered to indicate a planar binding mode of the ligand, where it is held fixed in a specific orientation with the protein’s active site. The similarity of these interactions implies that all four compounds bind to the target protein in a very similar manner, and these residues are critical in preserving the conformation of the ligand-protein complex. However, there are significant differences among the compounds that might affect the binding interactions. For example, CNP0079056 in the 50% interaction group interacts with TRP 219 in a manner different from others in the same group. This partial interaction indicates that TRP 219 may be involved in binding stability to a lesser extent but in a different mode of interaction that may allow the ligand to accommodate minimal structural variations in the binding pocket. This could prove advantageous in a fluctuating environment, where slight changes in the binding capacity could improve the compound’s affinity or specificity. Like in the previous case, CNP0338283 interacts with ASP 212 as well, but with 50% frequency. Such interaction could give an additional electrostatic contribution that adds further contact points, which are not always demanded but could give some additional contribution to the orientation of the ligand. However, the control ligand has a slightly different interaction mode to the test compounds. As it establishes close and persistent contact with the core residues at 100% frequency, it also has extra communication with GLU148 and ASP117 at 55% and 60%, respectively. Such interactions are not as persistent and as frequent as they are in the case of the core residues implying that the binding mode is more dynamic or less stable. The fact that these additional but partial interactions are relied upon by the control ligand might simply mean that it does not fit as securely into the binding pocket as the test compounds. Such partial interactions are identified in both the test compounds as well as the control molecules – the involvement of certain residues is more stable and uniform, while others contribute partially or only under specific conditions. The test compounds demonstrated consistent interactions with particular key residues and additional opportunities to form stabilizing contacts, which implies that the compounds may have a well-balanced binding process capable of effectively inhibiting the target protein.

#### 3.4.5 SASA analysis

**Figure 8:**
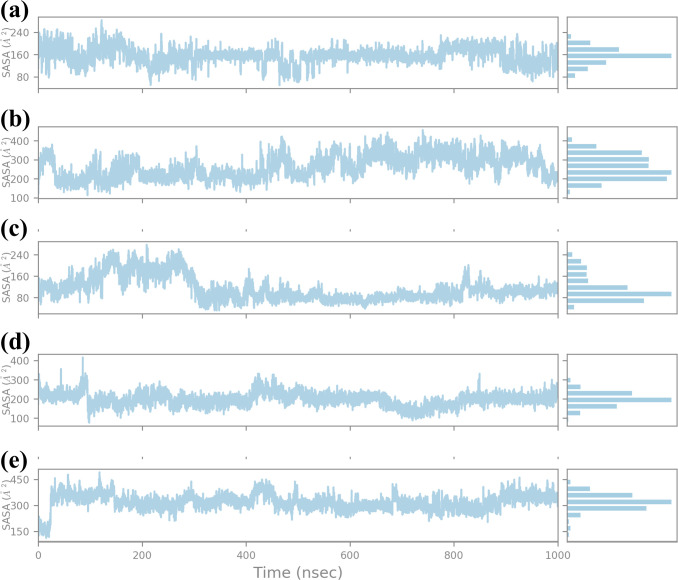
Ligand SASA plot obtained form the simulated VIM1-natural compounds **(A)** CNP0390322, **(B)** CNP0390569, **(C)** CNP0079056, **(D)** CNP0338283 and **(E)** XQ5 (reference).

SASA was calculated for a 1000 ns molecular dynamics (MD) simulation to compare the effect of four compounds on the structural stability and dynamics of the protein-ligand complex, as presented in **Figure 8**. SASA is another important feature that represents the solvent-exposed surface of the protein and provides information about the structural flexibility of the ligands in the binding pocket (Durham et al., 2009). SASA analysis for compound CNP0390322 has the range of about 160 Å²-240 Å² with average SASA value of approximately 200 Å². The plot shows that for a major part of the simulation, the complex keeps a relatively constant value of solvent exposure and only has small oscillations occasionally. The histogram indicates that most of the residues are near the midpoint of the histogram, which suggests that the protein-ligand complex exists most often in a stable conformation with reasonable solvation. Such stability shows that CNP0390322 binds nicely within the pocket and does not create a strain on the amino-acid residues, which could distort the conformation of the protein. Where CNP0390569 is concerned the SASA values are more volatile, varying between 200 Å² and 400 Å², with the average value being closer to 300 Å². The strengthened values of SASA point to the fact that the protein experiences greater conformational changes due to the binding of CNP0390569. The histogram suggests that the complex samples had multiple conformations during the simulation and were, therefore, wider. This means that CNP0390569 brings fluctuation within the protein possibly making it less stable to bind and more exposed to solvent than CNP0390322. The SASA values during the MD simulation for CNP0079056 show an initial drop and then start oscillating around 160 Å², and then after rising up to approximately 300 Å², the average SASA was found to be around 220 Å². These trends indicate the initial formation of stronger interactions between CNP0079056 and the protein in a more compact conformation compared to the other parts of the simulation process upon the formation of more solvent-exposed conformations. It is evident from the histogram that it has a major peak around the lower SASA values and another peak at higher SASA values due to conformational changes that occurred in the simulation period. This behavior may suggest an actual molecule interaction that starts with rigorous binding and then becomes more flexible and exposed to solvent. Arranging the protein structures with “CNP0338283” gives a SASA profile of the range of 200-300 Å², 250 Å² mean SASA. The plot shows trends like CNP0390569 but slightly lower overall SASA; therefore, the protein is somewhat more compact than the one from CNP0390569. The histogram distribution is again less spread out than the first one and has a peak at the average value; therefore, while flexibility is observed, the protein-ligand complex with CNP0338283 retains a relatively stable level of exposure to the solvent. This could mean the forming of higher or more stable binding forces. SASA analysis of the control molecule reveals that the value hovers at about 300–400 Å² and does not fluctuate much during the entire 1000 ns simulation. The histogram provides evidence of the stability of the protein in the absence of the tested compounds, as there is a relatively steep bell-shaped curve centered at the average value of the protein. These stable SASA values are expected as a foundation; therefore, the control molecule maintains the structural stability of the protein.

#### 3.4.6 RG analysis

**Figure 9:**
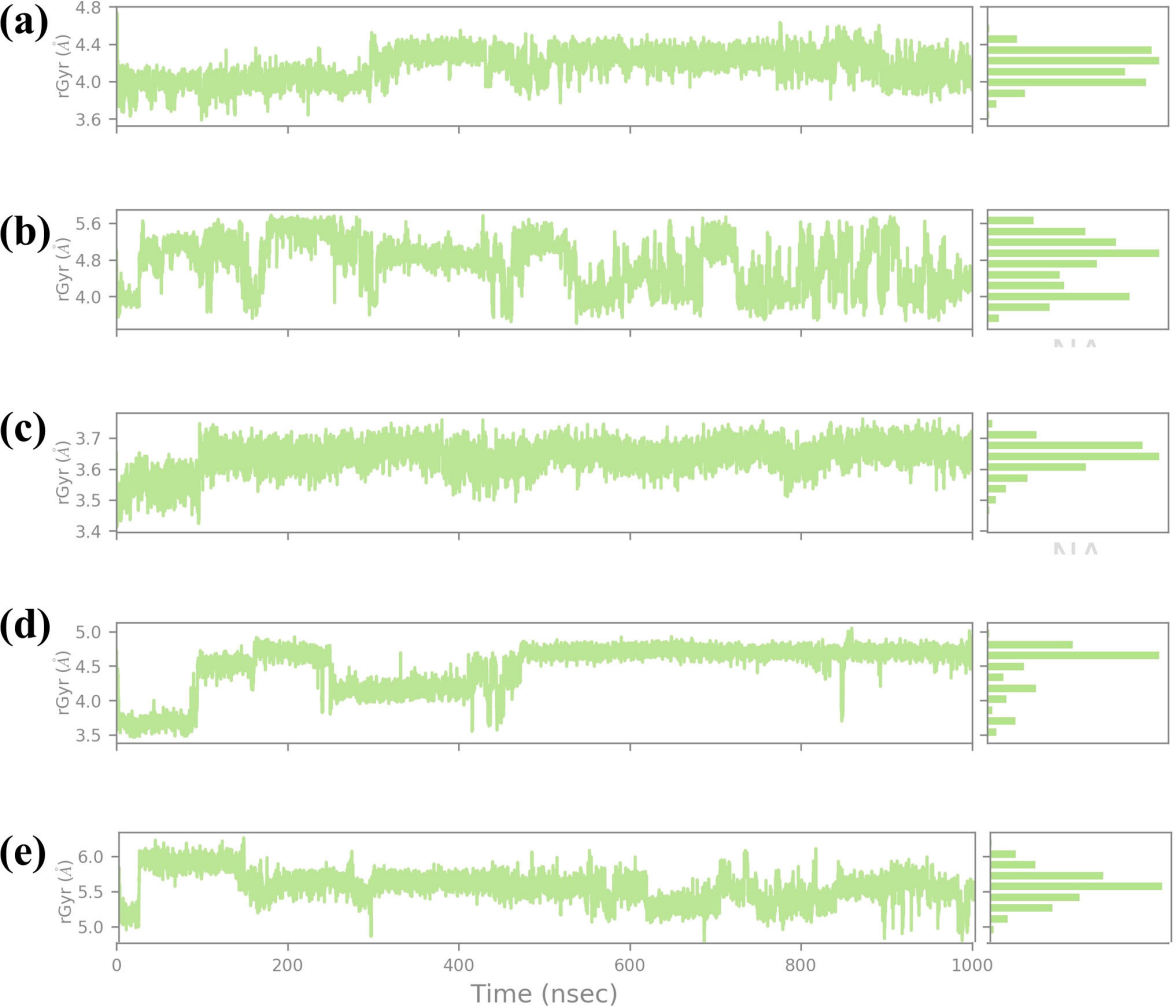
Ligand RG plot obtained form the simulated VIM1-natural compounds **(A)** CNP0390322, **(B** CNP0390569, **(C)** CNP0079056, **(D)** CNP0338283 and **(E)** XQ5 (reference).

The RG is a key parameter that provides information on the overall compactness of a protein structure during MD simulations. It is the depiction of the dispersion of atoms of the protein concerning the mass center of the protein. It is used for the determination of the conformational stability and folding propensities of the protein as described quantitatively in **Figure 9**. In this study, Rg values were analyzed to track the conformal stability of protein-ligand complexes with four chemical compounds and one control molecule up to 1000 ns simulation (Lobanov et al., 2008). The RG value of compound CNP0390322 measured between approximately 3.6 Å and 4.8 Å with a mean value of 4.2 Å. The changes in Rg values are not very significant throughout the simulation, which means that the protein-ligand complex remains nearly the same in terms of compactness. These minor variations are average for such kinds of simulations. However, the overall picture does not show that the binding of compound CNP0390322 induces any conformational changes in the protein.

The histogram is also constructed based on the Rg value, which is around the average Rg value, and the fact that this complex has a compact structure in the simulation process. For compound CNP0390569, the Rg profile is more spread out, ranging between 4.8 Å and 5.6 Å and an average of 5.2 Å. These fluctuations indicate that compound CNP0390569 causes more substantial conformational change that modifies the compactness of the protein. The broader distribution of the residues observed in the histogram may mean that the protein interacts with multiple conformations reflected as flexibility in binding to the compound CNP0390569. As seen from the simulation, the Rg values obtained are relatively higher against compound CNP0390322, which stalls that the protein-ligand complex is more expanded. The Rg values of compound CNP0079056 lie between about 3.4 Å to 3.7 Å but mostly are about 3.6 Å. The quite slight variation and the stable behavior of the Rg plot indicate that compound CNP0079056 keeps the protein relatively compact during the entire simulation. The images representing the delta Rg more or less resemble a normal distribution, though slightly skewed towards the left, and further suggest minimal conformational changes and highly stable interaction between compound CNP0079056 and the protein. Thus, the fact that Compound CNP0079056 has lower Rg values compared to compounds CNP0390322 and CNP0390569 indicates it promotes a more compact protein conformation. The Rg profile of CNP0338283 is slightly more volatile, varying between 3.5 Å – 5.0 Å and an average of 4.2 Å. The stage of interaction at which the system exhibits relatively high RG values and then falls into a more compact state at approximately 300 ns. This behavior indicates that CNP0338283 starts with a more significant protein conformation than what is observed as the simulation continues.

The figure shows histogram distribution approximately in two peaks, which means the protein-ligand complex oscillates between two conformations throughout the simulation. This regularity might represent an initial binding event that then switches to a more condensed conformational state. The control molecule in the Rg analysis shows fluctuation of between 3.6 Å to 4.8 Å with an average of 4.4 Å. The oscillation is low, and the spread in the histogram is also minimal, which indicates that the control molecule keeps the protein in an almost similar conformation as that observed with the CNP0390322. The stability in the Rg values also implies that the control molecule does not have a huge impact on the global conformation of the protein and thus maintains similar compactness throughout the simulation step. This stable Rg profile can be considered as a starting point, showing the impact of the tested compounds on protein conformation.

### 3.5 Analysis of MM/GBSA

This was done using MMGBSA analysis for four compounds: CNP0390322, CNP0390569, CNP0079056, as well as CNP0338283, and a control to compare their binding interactions with a target receptor and the results provided in **Supplementary Table S3**. The outcome of this computational approach is quantitative information about the binding affinity and stability of the ligand-receptor complexes, notably ΔVDWAALS, ΔEEL, ΔEGB, ΔESURF, ΔGGAS, ΔGSOLV, and ΔGtotal. Of all the compounds discussed in this work, CNP0390322 showed the best binding profile. The most negative van der Waals energy was determined for this compound (-46.02 ± 3.27 kcal/mol) which points toward the hydrophobic interactions of this compound with the receptor. These interactions are important for the fixation of the ligand within the binding pocket. Furthermore, CNP0390322 incurred an attractive electrostatic energy of -41.08 ± 3.90 kcal/mol, indicating large ionic or H-bonding interactions that should stabilize the complex. The ΔEGB for this compound was, however, relatively high at 121.25 ± 5.39 kcal/mol suggesting an energetic cost for desolvation in this binding event. Nevertheless, these penalties were offset by the gain in van der Waals and electrostatic contributions. The ΔESURF was -14.69 ± 1.93 kcal/mol, which also supports the fact that hydrophobic interaction is also involved in the binding process. The cumulative ΔGtotal of CNP0390322 was found to be -19.45 ± 14.50 kcal/mol, thus showcasing the highest binding affinity than the rest of the studied compounds. The compound CNP0390569 also showed maximum binding interactions as compared to other compounds in terms of electrostatic energy which was -72.80 ± 16.32 kcal/mol. This indicates that CNP0390569 makes strong ionic or polar contacts with the receptor, which is important in the binding process. In contrast, the van der Waals energy for this compound was slightly less favorable, reporting -27.77 ± 5.87 kcal/mol, suggesting less hydrophobic interactions than in CNP0390322. The polar solvation energy (ΔEGB) was also high at 106.31 ± 16.09 kcal/mol; however, the non-polar solvation energy (ΔESURF) was relatively low at -9.12 ± 3.53 kcal/mol which seems to indicate reduced overall hydrophobicity. The net gas phase energy (ΔGGAS) for CNP0390569 was -100.58 ± 22.20 kcal/mol because of the strong non-covalent interactions in the complex. However, the total binding free energy (ΔGtotal) was relatively low (-3.39 ± 41.83 kcal/mol), though certain positive contributions could be assigned to the solvation penalty. CNP0079056 also had a moderate binding that was characterized by a van der Waal energy of 30.53 ± 5.50 kcal/mol as well as an electrostatic energy of -63.86 ± 16.51 kcal/mol. These values suggest that both hydrophobic and electrostatic interactions are favorable but not of the same magnitude as CNP0390322. The polar solvation energy (ΔEGB) was calculated for CNP0079056, which had the lowest value of 92.66 ± 16.47 kcal/mol, meaning the lowest desolvation penalty and, therefore, being beneficial for binding. However, the non-polar solvation energy (ΔESURF) was relatively low at -9.59 ± 1.72 kcal/mol, which indicates relatively small hydrophobic effects. The total binding free energy (ΔGtotal) of CNP0079056 was found to be -11.33 ± 40.22 kcal/mol which portrayed moderate binding affinity of the compound. CNP0338283 displayed a binding profile similar to CNP0079056, with slightly more favorable van der Waals energy (-33.75 ± 4.36 kcal/mol) and non-polar solvation energy (-15.83 ± 3.29 kcal/mol). However, its electrostatic energy was less favorable at -52.79 ± 17.84 kcal/mol. The total ΔGtotal for CNP0338283 was -14.74 ± 42.71 kcal/mol, suggesting that this compound also has moderate binding affinity but may benefit from further optimization.

## 4 Discussion

This study, aimed to identify potential inhibitors of the metallo-beta-lactamase VIM-1 enzyme from *P. aeruginosa* using a comprehensive computational approach. The analysis encompassed virtual screening, re-docking, intermolecular interaction studies, molecular dynamics (MD) simulations, RMSD, protein RMSF, ligand RMSF, protein-ligand interaction profiling, Rg analysis, SASA analysis, and MMGBSA calculations. A control molecule was used as a baseline for comparison to evaluate the efficacy of each selected compound. Virtual screening of the COCONUT database led to the identification of four promising natural compounds based on their binding energies (Guido et al., 2008), which ranged from -9.91 kcal/mol CNP0390322 to -9.33 kcal/mol (CNP0338283). Re-docking was performed to validate the initial screening results and further refine the binding poses (Agu et al., 2023). All selected compounds demonstrated strong binding affinities, with energies ranging between -8.7 kcal/mol to -9.6 kcal/mol, compared to -10.7 kcal/mol for the control molecule. Among these, CNP0390322 emerged as the most potent inhibitor, forming a stable interaction network characterized by multiple hydrogen bonds with critical residues such as Asn210 and hydrophobic contacts with Phe62, Tyr67, and Trp219. Compound CNP0390569, while also interacting with Asn210, showed a lower number of hydrophobic interactions and relied on a π-cation interaction with His116, suggesting a less stable binding compared to Compound CNP0390322. Compound CNP0079056 exhibited unique multiple π-π stacking interactions, indicating a specific and stable binding mode, whereas Compound CNP0338283 showed additional hydrophobic contacts similar to Compound CNP0390569, indicating a potentially stable interaction within the enzyme’s active site. The control molecule, although forming an extensive hydrogen bonding network, displayed fewer hydrophobic interactions, suggesting weaker stabilization within the hydrophobic pocket (Chen et al., 2016). Intermolecular interaction analysis provided insights into the nature and strength of interactions between the ligands and the VIM-1 active site. Compound CNP0390322 demonstrated a strong interaction profile with robust hydrogen bonds and hydrophobic contacts that likely contributed to its stable binding. Compound CNP0390569, while forming key hydrogen bonds, showed fewer hydrophobic interactions and relied more on electrostatic interactions, suggesting a potentially less stable interaction compared to Compound CNP0390322. Compound CNP0079056, with its unique π-π stacking interactions, indicated a strong binding profile, while Compound CNP0338283, despite similar interaction types to Compound CNP0390569, demonstrated additional flexibility in its interactions, potentially enhancing its binding stability. The control molecule, while interacting with the protein through hydrogen bonding, which is so crucial for binding proper and stable interactions within the binding pocket, exhibited fewer hydrophobic contacts, and therefore, the structure within the pocket was less stabilized. These MD simulations proffered a kinetic view of the stability and dynamics of the protein-ligand complexes for a duration of 1000ns (Bagewadi et al., 2023). The RMSD analysis showed that CNP0390322 had the least deviation from the initial orientation and kept the most stable interaction with the protein throughout the simulation with protein RMSD values between 1.5 Å and 2.0 Å. Similar to CNP0390322, the ligand RMSD also decreased after an initial adjustment phase hence confirming that the binding of the ligand was consistent within the active site. CNP0390569, however, showed significant fluctuations in ligand RMSD, particularly in the early stages, suggesting a less stable interaction. Protein RMSD for CNP0390569 also exhibited variability, reflecting minor conformational adjustments during the binding process. RMSD values of CNP0079056 were like that of CNP0390322. Thus, the interaction was quite stable with no major impact on the protein structure. While compound CNP0338283 exhibits moderate oscillations in both protein and ligand RMSD values, the overall binding was stable. The control molecule continued to have lower protein RMSD and, therefore, appeared to be more rigid compared to the selected compounds; however, the ligand RMSD demonstrated the flexibility of this molecule, thus possibly indicating comparatively less stable complexation with the receptor. The flexibility of certain residues during the simulation was identified by using RMSF analysis of the protein complexes. Compound CNP0390322, flexibility analysis revealed that the structure exhibited moderate flexibility in the amino acid position 170-190, which imparts dynamic character that could prove useful in the stability of the ligand-binding domain. Notably, more conformational flexibility was observed with CNP0390569 binding, as evidenced by the lower RMSF values, suggesting a less conformable interannual interaction that may be stronger. The flexibility and potential to form stable complexes were comparable for CNP0079056 and CNP0390322, considering the RMSF values, whereas CNP0338283 demonstrated somewhat higher RMSF values to indicate moderate structural stability and flexibility. The detailed RMSF analysis of ligand flexibility showed that CNP0390322 had more conformational freedom around specific atoms which could be helpful for biology interactions. CNP0390569 demonstrated linear elevations in flexibility, especially in the central area, indicating flexibility in the binding pocket. The changes in the middle region of CNP0079056 were the most variable, which might represent regions that could change with the protein conformation, while CNP0338283 was most consistent, suggesting regions of dynamic interactions with the protein as the conformation changes. As for the control ligand, the fluctuation in the RMSF values was much higher, suggesting inferior binding affinity of the control ligand with the enzyme. Additional identification of the protein-ligand interactions, to some extent, helped the analysis of the stability and type of the interactions over the simulation period. The compound CNP0390322 showed very favorable hydrogen bonding interactions and high hydrophobicity, which may have led to its relatively stable and selective binding. Compound CNP0390569, which forms significant numbers of hydrogen bonds, seems to be anchored less well in the binding pocket due to fewer hydrophobic interactions. CNP0079056 demonstrated the interaction of chemical bonds primarily hydrogen bonds and hydrophobic interactions, while the chemical structure of CNP0338283 showed that although it is similar to CNP0390569 in interaction profile, it is more flexible and can improve the binding mode. The control molecule, while making important contacts, had a relatively less favorable binding affinity and relied on entropic factors and water-mediated associations. It was revealed that during the simulation, the protein-ligand complexes are relatively more compact according to the RG results (Lobanov et al., 2008). The compound CNP0390322 had a stable pattern of Rg, proving the compactness and robust binding ability throughout the experiment. Compound CNP0390569 presented more significant oscillation, indicating that its binding state is less stable and the protein conformation has more significant change. Compound CNP0079056 had the most stable Rg profile, suggesting that the protein was folded well and bound tightly to the ligand in the active site, whereas compound CNP0338283 fluctuated at the beginning and then became strikingly stable, which means conformational changes in the protein occurred before reaching a compact structure. The control molecule exhibited an undisrupted Rg profile similar to CNP0390322 but may have weaker interactions relative to the modified molecules. The analysis using SASA also gave data about the ligands’ structural flexibility and bonding strength to the solvent-exposed surface of the protein. Compound CNP0390322 maintained relatively constant SASA values, suggesting proper ligand fit within the binding cavity. Compound CNP0390569 exhibited greater variability, indicating more conformational changes and generally lower binding affinity. The initial structure of compound CNP0079056 was effectively bound to the protein region, and the binding gradually loosened over time; the structure of compound CNP0338283 was moderately flexible and tightly bound to the protein. The control molecule had a stable SASA level, suggesting that the protein structure remained nearly unchanged, but the ligand-binding affinity may have been less robust. According to the energy quantification of MMGBSA, the binding free energies of the complexes were established (Genheden and Ryde, 2015).

Of all the compounds, compound CNP0390322 had the lowest binding free energy, showing that it bound well with stable interactions within the site. Compound CNP0079056 also showed high-affinity binding, as evident with free energy values. However, compounds CNP0390569 and CNP0338283 employed less negative free energy value, implying less stability for the compounds in binding. The control molecule, which also demonstrated consistent interactions with the protein, had higher free energy values, pointing to a relatively less stable binding environment relative to the selected compounds. Prior research has pointed out that the molecule stability of hydrogen bonds and the compound-specific hydrophobicity Index are crucial factors in strong and specific protein-ligand interactions. Similar to our findings, Rohan Patil et al. (2010) highlighted the role of these interactions in stabilizing ligand binding in disease management (Patil et al., 2010). Moreover, the orientations of inhibitors to metallo-beta-lactamases indicate that polar and nonpolar interactions include π-π stacking interactions and the role of electrostatic attractions in stabilizing the inhibitors within the active site, comparable to Compounds 1 and 3, in this study.

## 5 Conclusion

In conclusion, our comprehensive computational research approach successfully identified potential small molecule inhibitors of metallo-beta-lactamase VIM-1 from *P. aeruginosa*. Through a detailed analysis incorporating virtual screening, re-docking, molecular dynamics simulations, and various computational metrics (RMSD, RMSF, protein-ligand interaction analysis, RG, SASA, and MMGBSA), we pinpointed natural compounds that exhibit promising inhibitory effects against VIM-1. Among the compounds analyzed, Compounds CNP0390322 and CNP0079056 stood out due to their strong binding energies, stable hydrogen bonding, and consistent hydrophobic interactions. These interactions were further corroborated by minimal structural fluctuations as observed in RMSD analyses. The binding poses, as represented by Rg values and SASA plots, indicated that these ligands maintained stable placements within the binding pocket throughout the simulation runs. Although other compounds, such as CNP0390322 and CNP0338283, also showed potential inhibitory activity, they exhibited higher conformational entropy and lower binding stability. The control molecule XQ5 while demonstrating stable interaction patterns, showed greater ligand flexibility and reduced binding stability compared to the selected compounds. Consequently, Compounds CNP0390322 and CNP0079056 emerged as the most promising structural analogues, exhibiting considerable therapeutic potential to inhibit VIM-1. These findings warrant further *in vitro* and *in vivo* investigations to validate the efficacy of these compounds in developing effective therapies to combat multidrug-resistant *P. aeruginosa* infections. Future studies should focus on confirming these computational predictions and exploring the therapeutic applications of these promising inhibitors in treating MDR bacterial infections.

## Data Availability

The original contributions presented in the study are included in the article/**Supplementary Material**. Further inquiries can be directed to the corresponding authors.
