# Supplementary material for: Overcoming beta-lactam resistance in Pseudomonas aeruginosa by targeting metallo-beta-lactamase VIM-1: a one-microsecond molecular dynamics simulation study
Source: Front Cell Infect Microbiol. 2025 Feb 4;15:1521391. doi: 10.3389/fcimb.2025.1521391 (PMC11832520; doi:10.3389/fcimb.2025.1521391)
Supplement: Supplementary file 1 [file Table1.docx]

Supplementary Material

| **S.no** | **Title** | **Docking score** |
| --- | --- | --- |
| 1 | CNP0390322 | -10.816 |
| 2 | CNP0390569 | -10.736 |
| 3 | CNP0079056 | -10.763 |
| 4 | CNP0338283 | -10.734 |
| 5 | CNP0203835 | -9.319 |
| 6 | CNP0369438 | -9.252 |
| 7 | CNP0395427 | -9.205 |
| 8 | CNP0165417 | -9.173 |
| 9 | CNP0273177 | -9.129 |
| 10 | CNP0000134 | -9.118 |
| 11 | CNP0389644 | -9.084 |
| 12 | CNP0287947 | -9.078 |
| 13 | CNP0368300 | -9.066 |
| 14 | CNP0376467 | -8.992 |
| 15 | CNP0254403 | -8.928 |
| 16 | CNP0073062 | -8.923 |
| 17 | CNP0395874 | -8.897 |
| 18 | CNP0431409 | -8.873 |
| 19 | CNP0405984 | -8.871 |
| 20 | CNP0391398 | -8.838 |
| 21 | CNP0156008 | -8.782 |
| 22 | CNP0082170 | -8.745 |
| 23 | CNP0390922 | -8.739 |
| 24 | CNP0146665 | -8.726 |
| 25 | CNP0391694 | -8.716 |
| 26 | CNP0183138 | -8.706 |
| 27 | CNP0185954 | -8.698 |
| 28 | CNP0015528 | -8.652 |
| 29 | CNP0082167 | -8.643 |
| 30 | CNP0312785 | -8.637 |
| 31 | CNP0331860 | -8.633 |
| 32 | CNP0325458 | -8.629 |
| 33 | CNP0108278 | -8.625 |
| 34 | CNP0277843 | -8.608 |
| 35 | CNP0389128 | -8.563 |
| 36 | CNP0290008 | -8.556 |
| 37 | CNP0394405 | -8.555 |
| 38 | CNP0395824 | -8.544 |
| 39 | CNP0296056 | -8.542 |
| 40 | CNP0083028 | -8.534 |
| 41 | CNP0390460 | -8.528 |
| 42 | CNP0406842 | -8.523 |
| 43 | CNP0462609 | -8.517 |
| 44 | CNP0257033 | -8.51 |
| 45 | CNP0238034 | -8.507 |
| 46 | CNP0333143 | -8.505 |
| 47 | CNP0211413 | -8.503 |
| 48 | CNP0379666 | -8.47 |
| 49 | CNP0405545 | -8.469 |
| 50 | CNP0393277 | -8.45 |
| 51 | CNP0140876 | -8.432 |
| 52 | CNP0134189 | -8.43 |
| 53 | CNP0395596 | -8.428 |
| 54 | CNP0255268 | -8.413 |
| 55 | CNP0317356 | -8.389 |
| 56 | CNP0167711 | -8.366 |
| 57 | CNP0223201 | -8.355 |
| 58 | CNP0200453 | -8.351 |
| 59 | CNP0250825 | -8.351 |
| 60 | CNP0284357 | -8.325 |
| 61 | CNP0213601 | -8.322 |
| 62 | CNP0119497 | -8.32 |
| 63 | CNP0476376 | -8.317 |
| 64 | CNP0394716 | -8.317 |
| 65 | CNP0291352 | -8.307 |
| 66 | CNP0350512 | -8.281 |
| 67 | CNP0240856 | -8.251 |
| 68 | CNP0430681 | -8.248 |
| 69 | CNP0276653 | -8.247 |
| 70 | CNP0360934 | -8.233 |
| 71 | CNP0073828 | -8.228 |
| 72 | CNP0238963 | -8.222 |
| 73 | CNP0371652 | -8.21 |
| 74 | CNP0018705 | -8.209 |
| 75 | CNP0164908 | -8.192 |
| 76 | CNP0396849 | -8.16 |
| 77 | CNP0311155 | -8.159 |
| 78 | CNP0422771 | -8.15 |
| 79 | CNP0261911 | -8.135 |
| 80 | CNP0280237 | -8.126 |
| 81 | CNP0071034 | -8.113 |
| 82 | CNP0397473 | -8.112 |
| 83 | CNP0370982 | -8.084 |
| 84 | CNP0372755 | -8.083 |
| 85 | CNP0444509 | -8.08 |
| 86 | CNP0397346 | -8.074 |
| 87 | CNP0387566 | -8.053 |
| 88 | CNP0404802 | -8.052 |
| 89 | CNP0394362 | -8.048 |
| 90 | CNP0395141 | -8.04 |
| 91 | CNP0392659 | -8.037 |
| 92 | CNP0112129 | -8.01 |
| 93 | CNP0451735 | -8.002 |
| 94 | CNP0399638 | -7.999 |
| 95 | CNP0385903 | -7.99 |
| 96 | CNP0258712 | -7.962 |
| 97 | CNP0287486 | -7.961 |
| 98 | CNP0396545 | -7.959 |
| 99 | CNP0011870 | -7.939 |
| 100 | CNP0440201 | -7.937 |
| 101 | CNP0266010 | -7.918 |
| 102 | CNP0348633 | -7.918 |
| 103 | CNP0324470 | -7.912 |
| 104 | CNP0368271 | -7.908 |
| 105 | CNP0369573 | -7.894 |
| 106 | CNP0184382 | -7.892 |
| 107 | CNP0395848 | -7.885 |
| 108 | CNP0109432 | -7.863 |
| 109 | CNP0277619 | -7.863 |
| 110 | CNP0118791 | -7.861 |
| 111 | CNP0179823 | -7.839 |
| 112 | CNP0391542 | -7.834 |
| 113 | CNP0391477 | -7.831 |
| 114 | CNP0149826 | -7.831 |
| 115 | CNP0369463 | -7.828 |
| 116 | CNP0232835 | -7.826 |
| 117 | CNP0136165 | -7.825 |
| 118 | CNP0009630 | -7.804 |
| 119 | CNP0300257 | -7.8 |
| 120 | CNP0396354 | -7.774 |
| 121 | CNP0419265 | -7.768 |
| 122 | CNP0384519 | -7.768 |
| 123 | CNP0334489 | -7.767 |
| 124 | CNP0476232 | -7.724 |
| 125 | CNP0352896 | -7.711 |
| 126 | CNP0438441 | -7.708 |
| 127 | CNP0120660 | -7.706 |
| 128 | CNP0237903 | -7.701 |
| 129 | CNP0394350 | -7.697 |
| 130 | CNP0394483 | -7.677 |
| 131 | CNP0326787 | -7.672 |
| 132 | CNP0393976 | -7.671 |
| 133 | CNP0141124 | -7.669 |
| 134 | CNP0352593 | -7.667 |
| 135 | CNP0417511 | -7.666 |
| 136 | CNP0396677 | -7.659 |
| 137 | CNP0004417 | -7.652 |
| 138 | CNP0369218 | -7.646 |
| 139 | CNP0239566 | -7.646 |
| 140 | CNP0235882 | -7.641 |
| 141 | CNP0358032 | -7.633 |
| 142 | CNP0337097 | -7.633 |
| 143 | CNP0122239 | -7.63 |
| 144 | CNP0315540 | -7.629 |
| 145 | CNP0387839 | -7.62 |
| 146 | CNP0231395 | -7.618 |
| 147 | CNP0160352 | -7.603 |
| 148 | CNP0383450 | -7.585 |
| 149 | CNP0177763 | -7.571 |
| 150 | CNP0366811 | -7.564 |
| 151 | CNP0200626 | -7.564 |
| 152 | CNP0363220 | -7.546 |
| 153 | CNP0337728 | -7.54 |
| 154 | CNP0012247 | -7.534 |
| 155 | CNP0246960 | -7.532 |
| 156 | CNP0293952 | -7.525 |
| 157 | CNP0375787 | -7.523 |
| 158 | CNP0357122 | -7.518 |
| 159 | CNP0010124 | -7.514 |
| 160 | CNP0396341 | -7.503 |
| 161 | CNP0397258 | -7.503 |
| 162 | CNP0145108 | -7.483 |
| 163 | CNP0373452 | -7.479 |
| 164 | CNP0196281 | -7.459 |
| 165 | CNP0397585 | -7.456 |
| 166 | CNP0228459 | -7.456 |
| 167 | CNP0466969 | -7.446 |
| 168 | CNP0038517 | -7.446 |
| 169 | CNP0387557 | -7.439 |
| 170 | CNP0401108 | -7.434 |
| 171 | CNP0108941 | -7.426 |
| 172 | CNP0219069 | -7.424 |
| 173 | CNP0312425 | -7.415 |
| 174 | CNP0139429 | -7.407 |
| 175 | CNP0370468 | -7.394 |
| 176 | CNP0165098 | -7.391 |
| 177 | CNP0403477 | -7.389 |
| 178 | CNP0284809 | -7.381 |
| 179 | CNP0014290 | -7.378 |
| 180 | CNP0391501 | -7.377 |
| 181 | CNP0289372 | -7.37 |
| 182 | CNP0124335 | -7.365 |
| 183 | CNP0236147 | -7.345 |
| 184 | CNP0281081 | -7.333 |
| 185 | CNP0033693 | -7.331 |
| 186 | CNP0293462 | -7.311 |
| 187 | CNP0376261 | -7.311 |
| 188 | CNP0427626 | -7.291 |
| 189 | CNP0461350 | -7.277 |
| 190 | CNP0279257 | -7.265 |
| 191 | CNP0366322 | -7.264 |
| 192 | CNP0396579 | -7.263 |
| 193 | CNP0179829 | -7.257 |
| 194 | CNP0402969 | -7.252 |
| 195 | CNP0316927 | -7.249 |
| 196 | CNP0451655 | -7.242 |
| 197 | CNP0229326 | -7.24 |
| 198 | CNP0396903 | -7.235 |
| 199 | CNP0459339 | -7.229 |
| 200 | CNP0370380 | -7.227 |
| 201 | CNP0395976 | -7.223 |
| 202 | CNP0396409 | -7.211 |
| 203 | CNP0401553 | -7.179 |
| 204 | CNP0396973 | -7.177 |
| 205 | CNP0119985 | -7.173 |
| 206 | CNP0283671 | -7.171 |
| 207 | CNP0267452 | -7.151 |
| 208 | CNP0212896 | -7.142 |
| 209 | CNP0010023 | -7.138 |
| 210 | CNP0243415 | -7.123 |
| 211 | CNP0424889 | -7.122 |
| 212 | CNP0148012 | -7.109 |
| 213 | CNP0018076 | -7.102 |
| 214 | CNP0247407 | -7.101 |
| 215 | CNP0353748 | -7.098 |
| 216 | CNP0376462 | -7.088 |
| 217 | CNP0346994 | -7.081 |
| 218 | CNP0039827 | -7.06 |
| 219 | CNP0435303 | -7.059 |
| 220 | CNP0011839 | -7.047 |
| 221 | CNP0010582 | -7.045 |
| 222 | CNP0364911 | -7.039 |
| 223 | CNP0396793 | -7.036 |
| 224 | CNP0395803 | -7.024 |
| 225 | CNP0370562 | -7.024 |
| 226 | CNP0345020 | -6.999 |
| 227 | CNP0378871 | -6.998 |
| 228 | CNP0292147 | -6.992 |
| 229 | CNP0011296 | -6.989 |
| 230 | CNP0471107 | -6.978 |
| 231 | CNP0015540 | -6.964 |
| 232 | CNP0318339 | -6.954 |
| 233 | CNP0178212 | -6.93 |
| 234 | CNP0410719 | -6.914 |
| 235 | CNP0010210 | -6.903 |
| 236 | CNP0402217 | -6.901 |
| 237 | CNP0280073 | -6.881 |
| 238 | CNP0035608 | -6.86 |
| 239 | CNP0305243 | -6.823 |
| 240 | CNP0427134 | -6.815 |
| 241 | CNP0157423 | -6.811 |
| 242 | CNP0344012 | -6.81 |
| 243 | CNP0268258 | -6.8 |
| 244 | CNP0133333 | -6.739 |
| 245 | CNP0221892 | -6.737 |
| 246 | CNP0183395 | -6.731 |
| 247 | CNP0217668 | -6.72 |
| 248 | CNP0464804 | -6.692 |
| 249 | CNP0384121 | -6.664 |
| 250 | CNP0181744 | -6.654 |
| 251 | CNP0344081 | -6.557 |
| 252 | CNP0366843 | -6.554 |
| 253 | CNP0348456 | -6.537 |
| 254 | CNP0205711 | -6.536 |
| 255 | CNP0138116 | -6.524 |
| 256 | CNP0217884 | -6.519 |
| 257 | CNP0379914 | -6.459 |
| 258 | CNP0129046 | -6.458 |
| 259 | CNP0152532 | -6.412 |
| 260 | CNP0395694 | -6.396 |
| 261 | CNP0014758 | -6.377 |
| 262 | CNP0344858 | -6.364 |
| 263 | CNP0014720 | -6.356 |
| 264 | CNP0166366 | -6.347 |
| 265 | CNP0287591 | -6.334 |
| 266 | CNP0387074 | -6.312 |
| 267 | CNP0102469 | -6.31 |
| 268 | CNP0105728 | -6.276 |
| 269 | CNP0206430 | -6.246 |
| 270 | CNP0429239 | -6.243 |
| 271 | CNP0004966 | -6.214 |
| 272 | CNP0113845 | -6.202 |
| 273 | CNP0012332 | -6.169 |
| 274 | CNP0350632 | -6.123 |
| 275 | CNP0257191 | -6.104 |
| 276 | CNP0466110 | -6.078 |
| 277 | CNP0375205 | -6.077 |
| 278 | CNP0328286 | -6.067 |
| 279 | CNP0004625 | -6.05 |
| 280 | CNP0158438 | -6.027 |
| 281 | CNP0173346 | -6.013 |
| 282 | CNP0369980 | -5.995 |
| 283 | CNP0322605 | -5.893 |
| 284 | CNP0448129 | -5.808 |
| 285 | CNP0366292 | -5.786 |
| 286 | CNP0157246 | -5.614 |
| 287 | CNP0259572 | -5.601 |
| 288 | CNP0218057 | -5.392 |
| 289 | CNP0295332 | -5.156 |
| 290 | CNP0300904 | -5.105 |
| 291 | CNP0211195 | -5.049 |
| 292 | CNP0398704 | -4.998 |
| 293 | CNP0315723 | -4.987 |
| 294 | CNP0014318 | -4.976 |
| 295 | CNP0228737 | -4.938 |
| 296 | CNP0423937 | -4.899 |
| 297 | CNP0439967 | -4.898 |
| 298 | CNP0315453 | -4.877 |
| 299 | CNP0436470 | -4.736 |
| 300 | CNP0344027 | -4.727 |
| 301 | CNP0312411 | -4.663 |
| 302 | CNP0354811 | -4.613 |
| 303 | CNP0433369 | -4.543 |
| 304 | CNP0123741 | -4.535 |
| 305 | CNP0357672 | -4.425 |
| 306 | CNP0352248 | -4.378 |
| 307 | CNP0400611 | -4.369 |
| 308 | CNP0281950 | -4.355 |
| 309 | CNP0328172 | -4.351 |
| 310 | CNP0155784 | -4.303 |
| 311 | CNP0301407 | -4.242 |
| 312 | CNP0260875 | -4.167 |
| 313 | CNP0472057 | -4.127 |
| 314 | CNP0426345 | -4.127 |
| 315 | CNP0386411 | -4.111 |
| 316 | CNP0434454 | -4.107 |
| 317 | CNP0127570 | -3.995 |
| 318 | CNP0132019 | -3.98 |
| 319 | CNP0335917 | -3.934 |
| 320 | CNP0248494 | -3.92 |
| 321 | CNP0427323 | -3.885 |
| 322 | CNP0168555 | -3.791 |
| 323 | CNP0229373 | -3.708 |
| 324 | CNP0156171 | -3.672 |
| 325 | CNP0342302 | -3.552 |
| 326 | CNP0467052 | -3.472 |
| 327 | CNP0291107 | -3.395 |
| 328 | CNP0169335 | -3.25 |
| 329 | CNP0187491 | -3.182 |
| 330 | CNP0015825 | -3.129 |
| 331 | CNP0119455 | -3.112 |
| 332 | CNP0012151 | 7 |

**Table S1** List of selected compounds used in virtual screening

| **S no.** | **Complex** | **H-Bond** | **Hydrophobic** | **π-π**  **stacking/**  **π-π cation*** |
| --- | --- | --- | --- | --- |
| 1 | CNP0390322 | Asn^210^ | Phe^62^, Tyr^67^,  Trp^87^, Cys^198^,  Ala^208^, Ala^212^,  Trp^219^ | His^240^ |
| 2 | CNP0390569 | Asn^210^ | Phe^62^, Tyr^67^,  Pro^68^, Trp^87^,  Cys^198^, Ala^208^,  Ala^212^ | His^116^ |
| 3 | CNP0079056 | Asn^210^ | Phe^62^, Tyr^67^,  Pro^68^, Trp^87^,  Cys^198^ | Phe^62^, Tyr^67^,  Trp^87^, His^240^ |
| 4 | CNP0338283 | Asn^210^ | Phe^62^, Tyr^67^,  Pro^68^, Trp^87^,  Cys^198^, Ala^212^ | His^116^ |
| 5 | Control | Glu^146^, Ser^207^,  Gly^209^, Asn^210^,  Asp^213^ | Phe^62^, Tyr^67^,  Trp^87^, Phe^115^,  Cys^198^, Ala^212^ | His^116^ |

**Table S2** – Intermolecular analysis of four selected compound in complex with the target protein

| Energy components/ Complexes | CNP0390322 | CNP0390569 | CNP0079056 | CNP0338283 | Control |
| --- | --- | --- | --- | --- | --- |
| Van der Waal energy (ΔVDWAALS) | -46.02±3.27 | -27.77±5.87 | -30.53±5.50 | -33.75±4.36 | -29.95±4.48 |
| Electrostatic energy(ΔEEL) | -41.08±3.90 | -72.80±16.32 | -63.86±16.51 | -52.79±17.84 | -123.21±13.05 |
| Polar solvation energy (ΔEGB) | 121.25±5.39 | 106.31±16.09 | 92.66±16.47 | 87.64±17.21 | 145.10±11.13 |
| Non-polar solvation energy (ΔESURF) | -14.69±1.93 | -9.12±3.53 | -9.59±1.72 | -15.83±3.29 | -6.23±2.06 |
| Net gas phase energy (ΔGGAS) | -87.10±7.17 | -100.58±22.20 | -94.40±22.02 | -86.55±22.20 | -153.17±17.54 |
| Net solvation energy (ΔGSOLV) | -106.56±7.32 | -97.18±19.63 | -83.06±18.20 | -71.80±20.50 | -138.87±13.19 |
| ΔG_total_ | -19.45±14.50 | -3.39±41.83 | -11.33±40.22 | -14.74±42.71 | -14.30±30.74 |

**Table S3 –** MMGBSA analysis of four selected compound in complex with the target protein

**
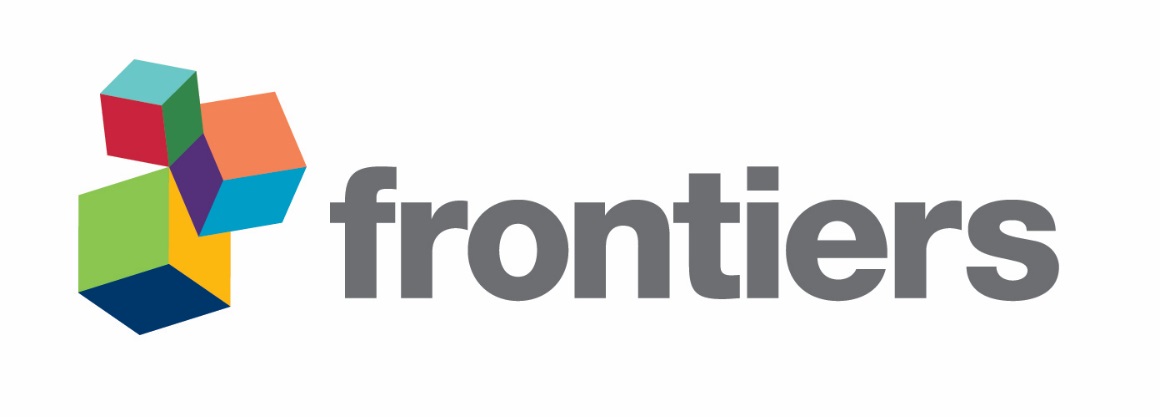
**

**Supplementary Figure 1.** The figure legends are required to have the same font as the main text, 12 point normal Times New Roman, single spaced. Please use a single paragraph for each legend and prepare the figures keeping in mind the PDF layout.
